# Silver acupuncture for myofascitis: A protocol for systematic review and meta-analysis

**DOI:** 10.1097/MD.0000000000020519

**Published:** 2020-06-05

**Authors:** Guilong Zhang, Yanming Lin, Qun Zhou, Liang Gao, Leixiao Zhang, Yang Yu, Yuquan Shen, Yong Huang

**Affiliations:** a Affiliated Hospital of Chengdu University of Traditional Chinese Medicine; b Chengdu University of Traditional Chinese Medicine, Chengdu, Sichuan; c Boai Hospital Affiliated to China Rehabilitation Research Center, Beijing; d Acupuncture and Tuina School, Chengdu University of Traditional Chinese Medicine; e The First People's Hospital of Long quanyi District, Chengdu, Sichuan, China.

**Keywords:** myofascitis, protocol, silver acupuncture, systematic review

## Abstract

**Background:**

This systematic review aims to evaluate the effectiveness and safety of silver acupuncture in treatment of myofascitis.

**Methods:**

Electronic databases of all silver acupuncture for myofascitis will be searched at PubMed, Cochrane Library, Springer, Embase, China National Knowledge Infrastructure, Wanfang, and Chinese Biological Medical disc from inception to March 31, 2020, with language restricted in Chinese and English. The primary outcome is visual analog scale, a short pain scale with sensitivity and comparability. Secondary outcomes included Clinical Assessment Scale for Cervical Spondylosis, Japanese Orthopaedic Association Scores, Oswestry dysfunction index, American Orthopaedic Foot and Ankle Society-Ankle Hindfoot scale, Foot and Ankle Ability Measure, The Cumberland ankle instability tool, Pittsburgh sleep quality index, self-rating anxiety scale, self-depression rating scale, and follow-up relapse rate. The systematic review and searches for randomized controlled trials of this therapy for myofascitis. The Cochrane RevMan V5.3 bias assessment tool is implemented to assess bias risk, data integration risk, meta-analysis risk, and subgroup analysis risk (if conditions are met). Mean difference, standard mean deviation, and binary data will be used to represent continuous results.

**Results:**

This study will provide a comprehensive review and evaluation of the available evidence for the treatment of myofascitis with this therapy.

**Conclusion:**

This study will provide new evidence to evaluate the effectiveness and side effects of silver acupuncture for myofascitis. Due to the data are not personalized, no formal ethical approval is required.

**Ethics and dissemination:**

There is no requirement of ethical approval and it will be in print or disseminated by electronic copies.

**PROSPERO registration number:**

CRD42020151476

## 1 Introduction

### 1.1 Description of the condition

Myofascitis, also known as myofascial pain symptom (MPS), is a common chronic musculoskeletal condition caused by chronic muscle strain and aseptic inflammation.^[1,2]^ Myofascial pain syndrome is a common source of musculoskeletal pain recognized by The International Association for the Study of Pain. The most common and specific symptom of myofascitis is referred and recurrent musculoskeletal pain that mainly affects the head, neck, shoulders, and lumbar muscles. Trigger points are defined as a pain generator and it is one of the most significant pathological features of MPS.^[1,3]^ This definition was first proposed by Travell and Simons^[4]^ in 1952 with 4 essential features as: tight bands, tenderness points, referred pain, and it could be alleviated by tender compression. Trigger points were confirmed existence by Simons and Travell^[5]^ in 1981 and discussed the diagnostic criteria for MPS.^[6]^ Clinically, trigger points have increasingly become a diagnostic method with clinical symptoms analysis and therapeutic targets of MPS.^[7]^

Trigger points are wildly used in different health care professions with different definitions. Myofascial trigger points are hyperirritable spots within a taut band of skeletal muscle that is painful on compression, stretch, overload, or contraction of the tissue which usually responds with a referred pain that is perceived distant from the spot.^[8]^ They are clinically classified as either active or latent. Active trigger points are defined by Simons et al as tender and spontaneous pain reported by patients which prevents full lengthening of the muscle, weakens the muscle, refers a patient-recognized pain, and mediates a local twitch response of muscle fibers when stimulated.^[9]^ The latent ones are also defined by Simons et al as quiescent pain and it is reproducible and stimulated when palpation occurs.^[9]^ Both of the 2 categories of trigger points induce common signs and symptoms of referred pain. A recent study has revealed that the trigger-point-associated pain symptoms may include different sensory sensations such as deep pain, distant pain, dull ache, tingling, burning sensation, and not just pain and proposed to replace the term of “referred pain ” with “referred sensation.”^[10]^

Etiology factors have been investigated in an attempt to understand how trigger points are associated with both sensory and motor symptoms of MPS. Myofascial trigger points may develop due to acute or repetitive muscle injuries or overloading, enthesopathies, joints injuries, poor posture, spine and intervertebral disc pathologies, and systemic diseases such as fibromyalgia.^[11,12]^ Some researches has proposed hypothesis that acetylcholine leakage may play a significant role during the process of MPS. Leakage of acetylcholine causes impairment of sarcoplasmic reticulum with following extensive calcium release, as well as secondary sarcomere contraction and cell membrane damage.^[8,13]^ Subsequently, the muscles remain in a high tension due to sustained contraction and result in ischemia and hypoxia. With the result of sustained contractive muscles leading to extensive calcium release, the vicious circle begins. Some studies show that inflammatory factors are contributive to MPS.^[14,15]^ Inflammatory cytokines, such as substance P, interleukin-1beta, tumor necrosis factor, and 5-hydroxytryptamin are found in a high level in the area of trigger-point muscles,^[16]^ and they are involved in the whole process of the pain mechanism.^[17]^ A recent study by Xie et al revealed that individuals with myofascial trigger-point-related chronic pain exhibited microstructural alterations in the gray matter, distributed in the limbic system and the pain matrix-associated brain areas.^[18]^

Interventions for MPS are aimed at relieving pain and improving mobility and function. The first-line choices for MPS are pharmacological treatment and noninvasive therapy.^[19]^ Nonsteroidal anti-inflammatory drugs (NSAIDs) are most commonly used to alleviate pain in most pain syndrome including MPS and it has mild side-effect to the digestive system. There are no randomized controlled trials (RCTs) specifically evaluating oral NSAIDs in the treatment of MPS though they are widely used in clinical practice. Therefore, there is a lack of strong evidence for the role of an anti-inflammatory in MPS.^[20]^ However, multiple studies demonstrated strong evidence in support of NSAIDs in the treatment of low back pain.^[21,22]^ Due to the considerable overlap between the MPS and low back pain, it is reasonable to consider NSAIDs have similar benefit in treating both pain syndrome. As it is mentioned above, the disadvantage is long-term use of NSAIDs may incur side effects to gastrointestinal, renal, and antiplatelet and doctors and patients both need to be cautious.^[23]^ Muscle Relaxants are used to decrease muscle spasticity. Tizanidine was documented by a study that it significantly decreases the pain intensity and disability from baseline, meanwhile improving the sleep duration and qualify.^[24]^ Due to its efficiency in the treatment of low back pain, some researchers also suggest tizanidine should be considered as a first-line agent to treat MPS.^[24]^ Anticonvulsants such as gabapentin and pregabalin have analgesic, anxiolytic-like, and anticonvulsant activity, which reduces the release of several neurochemicals, including glutamate, noradrenaline, and substance P.^[25]^ Two RCTs have evaluated the efficacy of tizanidine in patients with acute low back pain showed a significant difference in pain reduction that favored tizanidine over placebo.^[26,27]^ According to this, tizanidine may have similar therapeutic effect on MPS but there is still a lack of strong evidence. Cyclobenzaprine is the most widely studied anticonvulsants with efficiency for relieving skeletal muscle spasms and associating pain in acute musculoskeletal conditions.^[28]^ Two RCTs have evaluated the efficiency of cyclobenzaprine in patients with MPS.^[29,30]^ Another RCT study evaluated cyclobenzaprine found no evidence of such efficiency.^[31]^ With the aforementioned literature, there is also insufficient evidence supports the use of cyclobenzaprine in the treatment of MPS. There are many other pharmacological treatments like sedatives and hypnotics, neuropathic analgesics and antidepressants, but the main and first-choice treatment still has not reached a consensus.

There are numerous pharmacological treatments for MPS but simultaneously lack of satisfactory for both doctor and patients. Many patients choose to therapy and have to accept the side effects it brings. Currently, some complementary and alternative therapies drew more and more attention to treat MPS.

As one of the complementary and alternative therapies, traditional Chinese medicine (TCM) therapies play a vital role in preventing and treating many varieties of diseases including MPS. As one of TCM therapies, acupuncture has aroused lots of attraction from all over the world due to its efficiency in the clinical treatment.^[32]^

### 1.2 Description of the intervention

Acupuncture is one of the key components of TCM therapies in which thin needles are inserted into the body. It is developed and practiced in ancient China dating from Shang Dynasty (1600–1100 BC). Acupuncture is based on TCM theory in which health is deemed as keeping balance of energy that is called *Qi* flowing a circulation through the channels and meridians in the body. Acupuncture corrects the imbalance of the flowing of *Qi* via a needle inserted into special acupoints along the meridians.^[33]^ Acupuncture has been proved to be efficient in China for more than 2000 years and it had been widely used in treatment of varieties of diseases. Due to the efficiency of pain relief, it has increasingly drawn widespread attention and it was recommended by the World Health Organization since 1980 as an alternative therapy for 43 different disorders. An American survey found that 8 million Americans have been accepted acupuncture therapy in their lifetime and the most common used is for low back pain.^[34,35]^ According to recent studies, acupuncture can not only bring immediate pain relief but also improve the functional recovery of the whole body. Silver acupuncture is based on acupuncture which the material and shape is different from traditional acupuncture needle. The needles chosen for silver acupuncture therapy are longer and larger than traditional ones and it is composed with 80% of silver and other alloys. With the specific structure, silver acupuncture treatment has forged four characteristics. Firstly, it is inserted into the points attached to the skeletal muscle inserted of the acu-points, and trigger points are always chosen in the treatment of MPS. Secondly, silver needle is soft and it is not easily broken when pushing along the bony concave surface of the periosteum, which helps to enlarge the treating area and reach the accurate initial pain spot. Thirdly, silver needle is with diameter range from 1.0 to 1.1 mm, it is more likely to be intact and not easily broken at the hyper muscular contraction when needle is inserted into deep muscle tissue during the treatment. Lastly, it is a quick, simple, slight injury therapy for MPS. With the standard operation, silver acupuncture is safe to keep the stimulus under the endurance of the patients by keeping abreast of the patients’ needs.

### 1.3 How the intervention might work?

According to TCM theory, MPS is caused by *Qi* stagnation and blood stasis, external trauma or deficiency of *Qi* and blood, which all result in obstruction of *Qi* and blood flowing in the meridians. The principle of TCM treatment is to promote the circulation of *Qi* and blood of which relieving pain, activating the *Qi* stagnation and removing blood stasis, improving function. Silver acupuncture corrects the imbalance of the meridians through regulating *Qi* and blood and prolongs stimulation on accurate trigger points to relieve the pain immediately. Some modern medicine surveys found that MPS is treated by silver acupuncture may be through improving local microcirculation, adjusting the abnormal ultra-structure of nerve cell, regulating the immune system, decreasing the level of inflammatory cytokines, influencing the speed of nerve conduction velocity.^[36–39]^ There are many studies about how silver acupuncture works on MPS and some researchers came up with a variety of hypothesis; however, no consensus has been reached, leaving the main mechanism still remaining unclear.

### 1.4 Objectives

To develop treatment recommendations, we systematically evaluated the efficacy and safety of silver acupuncture for MPS.

## 2 Methods

### 2.1 Study registration

The PROSPERO registration number is CRD42020151476. This protocol report was performed according to the Preferred Reporting Items for Systematic Reviews and Meta-Analyses Protocols (PRISMA-P) statement guidelines.^[40]^ The review will be conducted in accordance with the PRISMA guidelines and follows the recommendations of the Cochrane Handbook for Systematic Reviews of Interventions.^[41,42]^ If we refine the procedures described in this protocol, we will update the record in the PROSPERO and disclose them in future publications related to this study.

### 2.2 Inclusion criteria for study selection

#### 2.2.1 Types of study

To evaluate the efficacy of silver acupuncture in the treatment of MPS, this paper only reviewed the RCT between silver acupuncture and the control group, including drug therapy, no treatment, placebo, diet, physical therapy, etc. In addition, both Chinese and English publications are restricted languages to this review. All RCTs that are not subject to publication state constraints will be included. If the experiment shows that the phrase is random and the blind method is not restricted, it will be regarded as a random study. Animal mechanism studies, case reports, self-controlled, non-RCTs, random crossover studies, and quasi-randomized trials will be excluded.

#### 2.2.2 Types of participants

Regardless of gender, age, ethnicity, education, and economic status, patients with MPS who meet the diagnostic criteria.^[43,44]^

#### 2.2.3 Types of intervention

The experimental group should be treated with silver acupuncture and trigger points used according to patients’ clinical manifestations and feedback. The types of seed used and the duration of treatment will be unlimited. Silver acupuncture combined with other conventional therapy should be excluded.

The following treatment comparisons will be investigated:

1. Silver acupuncture compared with no treatment
2. Silver acupuncture compared with placebo or sham treatment
3. Silver acupuncture compared with other active therapies
4. Silver acupuncture in addition to active therapy compared with the same active therapy

We will assess and compare the silver acupuncture according to how the acupuncturists have been trained and educated, on their clinical experience, on total numbers of silver acupuncture sessions, and on the treatment duration and frequency.

#### 2.2.4 Types of outcome measures

The primary outcome of this review was body function which is preferentially extracted from visual analog scale. Secondary outcomes include cervical spondylosis, Japanese Orthopaedic Association Scores, Oswestry dysfunction index, American Orthopaedic Foot and Ankle Society-Ankle Hindfoot scale, Foot and Ankle Ability Measure, The Cumberland ankle instability tool, Pittsburgh sleep quality index, Self-rating anxiety scale, self-depression rating scale, Pittsburgh sleep quality index, recurrence rate during the follow-up period, and adverse events.^[44,45]^ The system review will be performed independently.

### 2.3 Data sources

Our systematic review will search all RCTs of silver acupuncture for MPS electronically, regardless of publication status and language, by March 31, 2020. Databases include: PubMed, Embase, Springer, Web of Science, Cochrane Library, WHO International Clinical Trials Registry Platform (ICTRP), Traditional Chinese Medicine databases, China National Knowledge Infrastructure, China Biomedical Literature Database, Chinese Scientific Journal Database (VIP), and Wan-Fang database. Other sources, including reference lists of identified publications and meeting minutes, will also be searched.

### 2.4 Search strategy

The search strategy will be followed the PRISMA guidelines. The key search terms are (“myofascitis” OR “myofascial pain syndrome” OR “cervicoshoulder myofascitis” OR “lumbar myofascitis”) AND (“silver acupuncture” OR “silver needle”) AND (“randomized”). The search strategy will be adapted to different databases demands. Search strategy in PubMed is shown in Table 1.

**Table 1:**
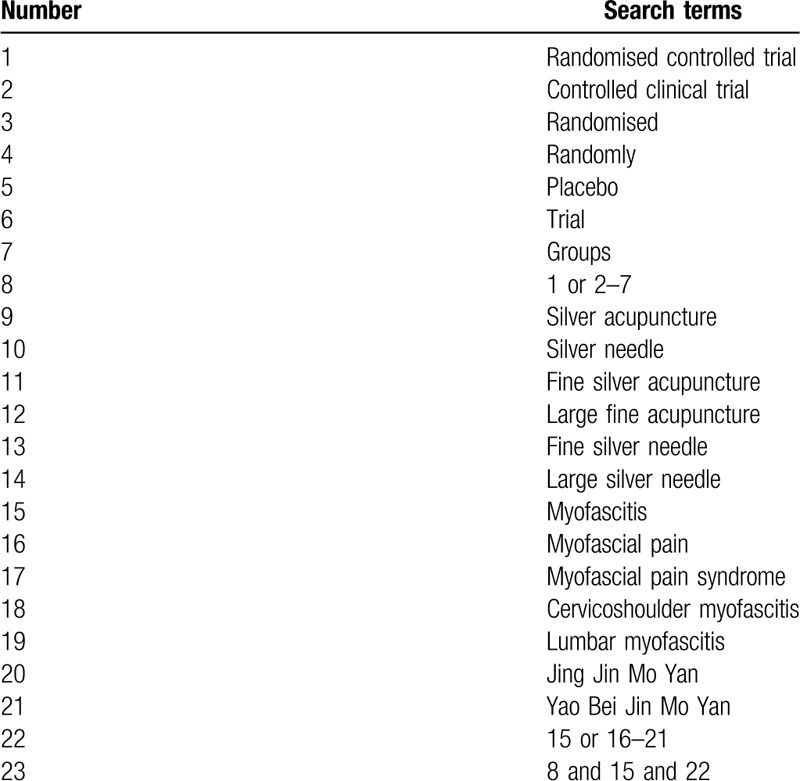
PubMed search strategy.

### 2.5 Data collection and analysis

#### 2.5.1 Selection of studies

**Figure 1:**
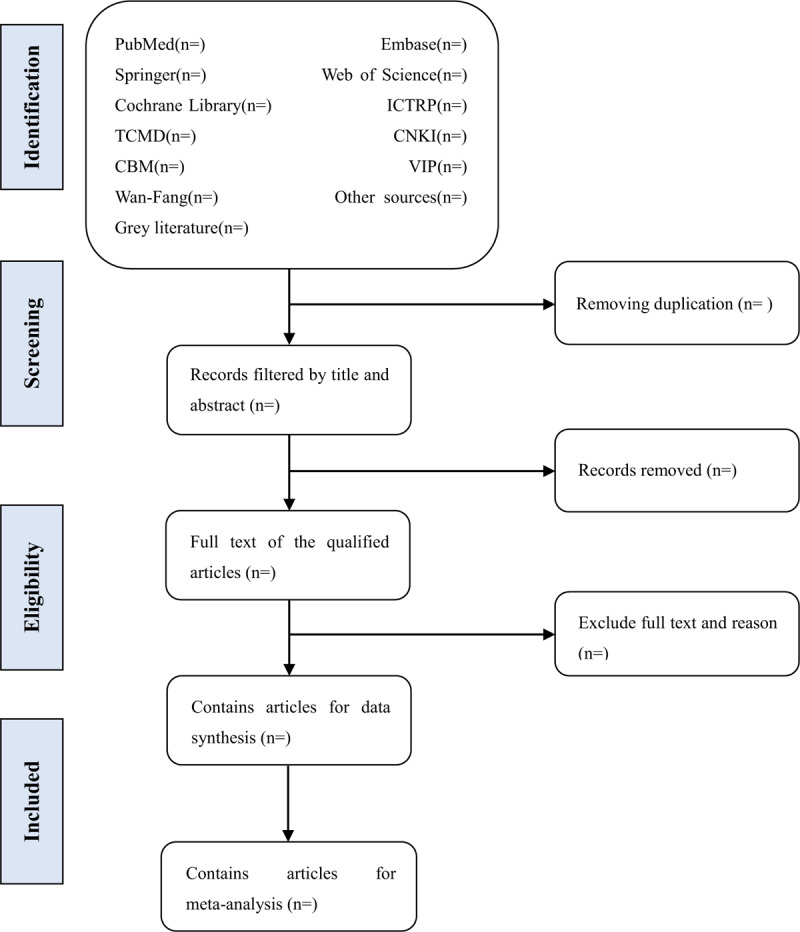
Flow diagram of studies identified.

Prior to literature retrieval, all reviewers are trained to ensure a basic understanding of the background and purpose of the review. In the literature screening process, we will use EndNote software (V.X8) document management software. The 2 comment authors (GLZ and LG) will be in strict accordance with the inclusion criteria, independent screen all retrieval research, read the title, abstract and key words in the literature, and determine which meet the inclusion criteria. We will obtain the full text of all relevant studies for further evaluation. Excluded studies will be documented and explained. If there is a disagreement in the selection process, it will be discussed by the 2 authors (GLZ and LG) and the 3rd author (LXZ) and arbitrated if necessary. If necessary, we will contact the trial author for clarification. The primary selection process is shown in a PRISMA flow chart (Fig. 1).

#### 2.5.2 Data extraction and management

The authors will extract data independently from the selected report or study and fill out the data extraction form. We will obtain data on general information, participants, methods, interventions, outcomes, results, adverse events, conflicts of interest, ethical recognition, and other information. For publications with insufficient or ambiguous data, we will attempt to obtain information from the corresponding authors by e-mail or telephone. Any differences will be resolved through discussions between the 2 authors, and further differences will be arbitrated by the 3rd author (LXZ).

#### 2.5.3 Assessment of risk of bias and reporting of study quality

The authors (LXZ and YQS) will use the Cochrane Collaboration's bias risk assessment tool to assess the risk of bias in all included studies. We will assess the risk of bias in the following areas: sequence generation, assignment sequence hiding, the blindness of participants and staff, and result evaluators, incomplete outcome data, selective outcome reporting, and other sources of bias. This review uses L, U, and H as the key to these assessments, where L (low) indicates a lower risk of bias, U (unclear) indicates that the risk of bias is uncertain, and H (high) indicates a higher risk of bias. If inconsistent results appear, the final decisions will be made by the 3rd author (YML). Information on the risk of biased assessments included in the study is summarized in tabular form and the results and impacts are critically discussed. If the information is ambiguous, we will try to contact the author. For repeated publications, we only select the original text.

#### 2.5.4 Measures of treatment effect

Data analysis and quantitative data synthesis will be performed using RevMan V.5.3. For continuous data, if there is no heterogeneity, we will use mean difference or standard mean deviation to measure the therapeutic effect of 95% confidence interval (CI). If significant heterogeneity is found, a random-effects model will be used. For dichotomous data, we will use the 95% CI risk ratio for analysis.

#### 2.5.5 Unit of analysis issues

We will include data from parallel group design studies for meta-analysis. Only the first phase of the data will be included in the random crossover trial. In these trials, participants were randomly divided into 2 intervention groups and individual measurements for each outcome of each participant were collected and analyzed.

#### 2.5.6 Management of missing data

If the primary result has missing or incomplete data, we will contact the author of the communication to obtain the missing data. If it is never available, exclude the experiment from the analysis.

#### 2.5.7 Assessment of heterogeneity

We will use the RevMan to assess efficacy and publication bias (version 5.3, Nordic Cochrane Centre, Copenhagen, Denmark). The forest map is used to illustrate the relative strength of the effect. The funnel plot is used to illustrate the bias because the number of trials exceeds 10. If a significant difference is detected, a random effects model will be used.

#### 2.5.8 Assessment of reporting biases

We will use a funnel plot to detect report bias. If more than 10 trials are included, the funnel plot will be used to assess the reported bias. If the funnel plot is found to be asymmetrical, analyze the cause using Egger method. We will include all eligible trials regardless of the quality of the method.

#### 2.5.9 Data synthesis

We will use RevMan for all statistical analysis. If considerable heterogeneity is observed, a 95% CI random-effects model will be used to analyze the combined effect estimates. Subgroup analysis will be performed with careful consideration of each subgroup if necessary.

#### 2.5.10 Subgroup analysis

There is no presubgroup plan. Subgroup analysis was performed based on control interventions and different outcomes.

#### 2.5.11 Sensitivity analysis

Based on sample size, heterogeneity quality, and statistical models (random- or fixed-effects models), we will perform sensitivity analysis.

#### 2.5.12 Grading the quality of evidence

The quality of evidence for all outcomes will be judged by the Grading of Recommendations Assessment, Development, and Evaluation working group approach. Bias risk, consistency, directness, precision, and publication bias are aspects of our assessment. High, medium, low, or very low represents the 4 levels of evaluation.^[46–48]^

## 3 Discussion

Pain is one of the most common reasons for an individual's visit to the outpatient clinic, and myofascial pain syndrome is diagnosed in nearly a 3rd of those who have musculoskeletal pain disorders. It is the main contributor to pain syndrome which affects a large population suffering all around the world. Despite the various of pharmacotherapies for MPS, numerous patients are still not satisfied with the therapeutic effect. And complementary treatment should be taken in consideration to remedy the deficiencies.

The evaluation of this systematic review will be divided into 4 parts: identification, the inclusion of literature, data extraction, and comprehensive analysis of data. According to the Cochrane method, this study is based on the analysis of clinical RCT evidence at home and abroad, searching, and screening the main electronic literature database with evidence-based medical evidence, providing clinicians with more convincing evidence in decision-making, to better guide clinical treatment.

## Author contributions

**Conceptualization:** Guilong Zhang, Yong Huang.

**Methodology:** Yanming Lin, Yuquan Shen, Qun Zhou.

**Software:** Liang Gao.

**Supervision:** Leixiao Zhang.

**Validation:** Yang Yu.

**Writing – original draft:** Guilong Zhang, Leixiao Zhang, Liang Gao, Yuquan Shen, Yang Yu.
